# Iron Oxide Nanoparticles as a Potential Iron Fertilizer for Peanut (*Arachis hypogaea*)

**DOI:** 10.3389/fpls.2016.00815

**Published:** 2016-06-09

**Authors:** Mengmeng Rui, Chuanxin Ma, Yi Hao, Jing Guo, Yukui Rui, Xinlian Tang, Qi Zhao, Xing Fan, Zetian Zhang, Tianqi Hou, Siyuan Zhu

**Affiliations:** ^1^College of Resources and Environmental Sciences, China Agricultural UniversityBeijing, China; ^2^College of Agriculture, Guangxi UniversityNanning, China; ^3^Stockbridge School of Agriculture, University of Massachusetts, AmherstMA, USA; ^4^Dow Pharma and Food Solution, The Dow Chemical Company, MidlandMI, USA

**Keywords:** peanut, Fe_2_O_3_ NPs, iron fertilizer, EDTA iron (iii), soil

## Abstract

Nanomaterials are used in practically every aspect of modern life, including agriculture. The aim of this study was to evaluate the effectiveness of iron oxide nanoparticles (Fe_2_O_3_ NPs) as a fertilizer to replace traditional Fe fertilizers, which have various shortcomings. The effects of the Fe_2_O_3_ NPs and a chelated-Fe fertilizer (ethylenediaminetetraacetic acid-Fe; EDTA-Fe) fertilizer on the growth and development of peanut (*Arachis hypogaea*), a crop that is very sensitive to Fe deficiency, were studied in a pot experiment. The results showed that Fe_2_O_3_ NPs increased root length, plant height, biomass, and SPAD values of peanut plants. The Fe_2_O_3_ NPs promoted the growth of peanut by regulating phytohormone contents and antioxidant enzyme activity. The Fe contents in peanut plants with Fe_2_O_3_ NPs and EDTA-Fe treatments were higher than the control group. We used energy dispersive X-ray spectroscopy (EDS) to quantitatively analyze Fe in the soil. Peanut is usually cultivated in sandy soil, which is readily leached of fertilizers. However, the Fe_2_O_3_ NPs adsorbed onto sandy soil and improved the availability of Fe to the plants. Together, these results show that Fe_2_O_3_ NPs can replace traditional Fe fertilizers in the cultivation of peanut plants. To the best of our knowledge, this is the first research on the Fe_2_O_3_ NPs as the iron fertilizer.

## Introduction

With the rapid development of nanotechnology, nanomaterials are increasingly used in the fields of aerospace, environment, industry, and agriculture (Le et al., 2014). Nanomaterials consist of nanometer-scale particles with a very small diameter and a large specific surface area. Compared with traditional materials, nanomaterials have many special functions resulting from the quantum size effect, macroscopic quantum tunneling, and dielectric confinement effect (Michalet et al., 2005; Hochella et al., 2008), and also have some new functions (Gui et al., 2015a). Consequently, such nanomaterials have many potential applications.

In previous studies, nanomaterials have been shown to promote seed germination, enhance degradation of pesticide residues, and improve soil quality (Ghrair et al., 2010; Khot et al., 2012; El-Temsah et al., 2014). In September 2003, the United States Department of Agriculture stated the importance of using nanotechnology in agricultural production; since then, many other countries have increased research efforts in this field (Sanguansri and Augustin, 2006). In addition, many experts have proposed that nanoparticles should be used in the field of soil–plant nutrition to achieve sustainable development of agricultural production with minimal environmental impacts (Sastry et al., 2007). Nanotechnology is a new technical revolution. Therefore, nanomaterials will become the new material for agricultural development, and represent new ideas and directions for global agricultural production (Chen and Yada, 2011).

Iron (Fe) is an essential nutrient for all organisms (Zuo and Zhang, 2011). Iron deficiency is widespread among many different crops, and peanut (*Arachis hypogaea*) is particularly sensitive (Sánchez-Alcalá et al., 2014). Fe content in soil is usually high, but a large proportion is fixed to soil particles (Mimmo et al., 2014; Bindraban et al., 2015). Fe is mainly in the form of insoluble Fe^3+^, especially in high-pH and aerobic soils; therefore, these soils are usually deficient in the available form, Fe^2+^ (Ye et al., 2015). Because plants usually absorb Fe^2+^ from soil, Fe-deficient soils lead to Fe-deficient plants (Kobayashi and Nishizawa, 2012). In plants, Fe participates in many physiological processes including chlorophyll biosynthesis, respiration, and redox reactions (Mimmo et al., 2014; Ye et al., 2015; Zargar et al., 2015). Also, considering the soil–plant–animal–human food chain, Fe deficiency not only affects the growth and development of plants, but can also lead to anemia in animals and humans (Guerinot and Yi, 1994; Li et al., 2014). Therefore, it is important to improve the utilization efficiency of Fe fertilizers. Peanut is an important oil and cash crop in China, and in most of its cultivation area the soil texture is gravely and sandy (Ding et al., 2011). Peanut is extremely sensitive to Fe deficiency, especially in alkaline soils (Zuo and Zhang, 2011). In calcareous soils, less than 10% of the Fe is available to plants (Mortvedt, 1991). Therefore, Fe deficiency is an important factor that can decrease crop yield and lower the quality of the peanut crop. The application of Fe fertilizer is still the most effective method to improve Fe deficiency in plants. The common varieties of Fe fertilizers include inorganic-Fe fertilizer, chelated-Fe fertilizer, and organic-Fe fertilizer (Laurie et al., 1991). Chelated-Fe fertilizer is more expensive, and is often applied to high-value crops. Soluble inorganic-Fe fertilizer does little to improve the available Fe content in alkaline calcareous soils. Organic-Fe fertilizer is readily adsorbed onto soil particles, which can reduce the fertilizer effect. Therefore, it is often used in soilless cultivation and as a foliar spray (Cesco et al., 2000; Lucena et al., 2010).

We want to find a new type of fertilizer that can solve the deficiencies of traditional fertilizers. Recently, the use of nanomaterials in agricultural production has increased (Gogos et al., 2012; Bindraban et al., 2015). Previous studies have shown that nanomaterials have potential applications as crop fertilizers because of their physical and chemical attributes (Bindraban et al., 2015; Montalvo et al., 2015). Iron oxide nanoparticle (Fe_2_O_3_ NP) is one of the most important oxides in the field of nanomaterials. Fe_2_O_3_ NPs have been widely applied in catalysis, magnetic materials, biomedicine, water treatment, and other fields (Cheng et al., 2015, 2016). The study found that Fe_2_O_3_ NPs can be uptake and transport by watermelon plants (Li et al., 2013). To date, there have been few reports on the use of nanomaterials as fertilizers for agricultural production, and all of them focused on ideal hydroponic conditions, rather than field conditions. Several studies on Fe_2_O_3_ NPs focused on the toxicity of nanomaterials (Burke et al., 2014; Li et al., 2014; Mattiello et al., 2015; Nhan et al., 2015; Rui et al., 2015). Research on the use of Fe_2_O_3_ NPs as a fertilizer still lags behind. Therefore, we investigated the effects of Fe_2_O_3_ NPs on the growth and Fe efficiency of peanut plants under field conditions to evaluate their use as an new Fe-fertilizer.

## Materials and Methods

### Fe_2_O_3_ NPs and Transmission Electron Microscope Characteristics

**FIGURE 1:**
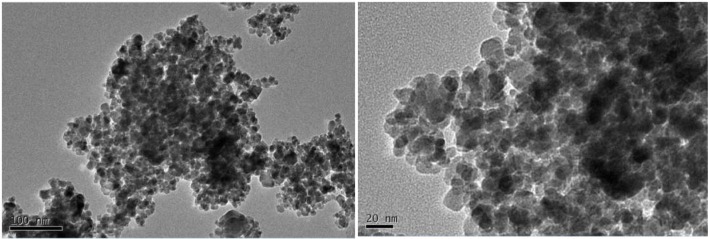
**Transmission electron microscope image of Fe_2_O_3_ NPs**.

In this study, the iron oxides nanometer materials are maghemite (γFe_2_O_3_). The Fe_2_O_3_ NPs with 20 nm average particle size were purchased from Shanghai Pantian Powder material Co., ltd. The shape and size were determined by transmission electron microscope (TEM) from Tsinghua University as shown in **Figure 1**. According to the information provided by the manufacturer, majority of the nanoparticles had spherical morphology and the diameter size ranged from 10 to 50 nm.

### Germination and Growth Conditions of Peanut Seeds

Peanut (*Arachis hypogaea*) seeds of variety ‘Kainong 15’ were obtained from Kaifeng Academy of Agricultural Sciences. The seeds were sterilized in 5% hydrogen peroxide solution for 10 min followed by three times rinsing with deionized water, and subsequently soaked in 50°C of deionized water for 4 h. The seeds were germinated in petri dishes covered with wet filter paper and placed in an incubator at 25 ± 1°C till the sprouts were approximately 1 cm length. The seeds of uniform size were selected to planting. Six peanut seeds were sowed in each pot and five replicates were applied in each treatment. After 38 days, three healthy seedlings with similar size were kept in each pot till harvest.

### Soil Preparation

The soils were collected from Shangzhuang experimental station of China Agricultural University. The basic physical and chemical properties of the soils were as following: Inorganic nitrogen: 13.88 mg kg^-1^, available phosphorus: 2.55 mg kg^-1^, available potassium: 56 mg kg^-1^, organic matter: 4.83 mg kg^-1^, available iron: 6.56 mg kg^-1^, pH 8.1. The soils were air dried then sieved through a 2 mm mesh. Dry sand was added to the soil, sand was applied in 1:5.5 ratio with soil (Priester et al., 2012; Ge et al., 2014), and applied fertilizer at the rate of N:P_2_O_5_:K_2_O = 0.25:0.3:0.25 mg kg^-1^. Each pot (inside dimension: 14 cm, height: 13.5 cm) filled in 1.5 kg mixture with different (0, 2, 10, 50, 250, and 1000 mg kg^-1^) concentrations of Fe_2_O_3_ NPs and 45.87 mg kg^-1^ EDTA iron (Liu et al., 2012). After germination, only the three strongest seedlings were allowed to grow. Pot experiment was conducted under the greenhouse conditions in West Campus China Agricultural University.

### Chlorophyll Measurement

The chlorophyll concentrations were measured using the Konic Minolta SPAD-502 Plus in the first fully expanded leaves of each plant. Five points were measured at different positions across the same leaf, and measured nine peanuts per treatment.

### Antioxidant Enzymes and Malondialdehyde (MDA) Assay

Antioxidant enzymes activity and MDA concentration were measured using the different enzyme-linked immunosorbent assay (ELISA) Kit purchased from Nanjing Jiancheng Bioengineering Institute (Nanjing, China). After 38 days from sowing, the harvested plants were separated into roots and shoots, and used for enzymatic analysis. Samples were grinded with four times volume of hydrochloric acid used a mortar and pestle under low temperature condition. The homogenate was centrifuged at 3500 rpm (3 k1s, SIGMA, USA) for 10 min at 4°C, then take the supernatant in accordance with the manual operation. All spectrophotometric analyses were used by 2800 UV/VIS (UNICO, Shanghai).

### Phytohormone Measurement

The phytohormones of gibberellin (GA4+7, GA3), zeatin-riboside (ZR), dihydrozeatin (DHZR), and indolepropionic acid (IPA) were analyzed by ELISA (Thermo, MULTISKAN MK3). After the harvest of peanuts, surface was washed clean, weighed (0.5 g ± 0.01), triturated, extracted, purified, and quantified by ELISA as described in Wang et al. (2012). The reagents and antibodies provided by Professor B Wang (China Agricultural University, Beijing, China).

### Biomass and Total Iron Content Determination

After 38 days from sowing, the harvested plants were separated into roots and shoots. The plant height, root length, and the number of branches were measured. The roots and shoots were deactivation of enzymes for 30 min at 105°C then at 75°C until the materials reached a constant weight. The dry tissues were weighed and ground into powder to measure the content of total iron. The samples were digested with the mixed solutions of HNO_3_-HF (1:2 ratio) for 24 h in a Single Reaction Chamber Microwave Digestion System (MILESTONE, LabTech, Vergamo, Italy). Then, the acid mixture was evaporated on an electric plate (VB20, LabTech) at 210°C until the solution reduced to 1 ml, and then the residue was diluted with ultrapure water. The total iron content was measured by inductively coupled plasma optical emission spectrometry (ICP-OES; ICAP 6300, Thermo Scientific, Waltham, MA, USA).

### Scanning Electron Microscope Characterization of Iron in Soil

The irons in soil were analyzed using scanning electron microscope (SEM) equipped with an Energy Dispersive X-ray Spectroscopy (EDS). The acceleration voltage is 15 kV. The control and 1000 mg⋅kg^-1^ treated were determined in this study.

### Statistical Analysis

All results were conducted with three replicates. The experimental data were statistically evaluated by one-way analysis of variance (ANOVA) utilized the SPSS 19 and Excel software, and the mean values for each treatment were compared using the Duncan’s test at the *P* < 0.05 confidence level.

## Results

### Effect of Fe_2_O_3_ NPs on Biomass, Plant Height, and Number of Branches

**FIGURE 2:**
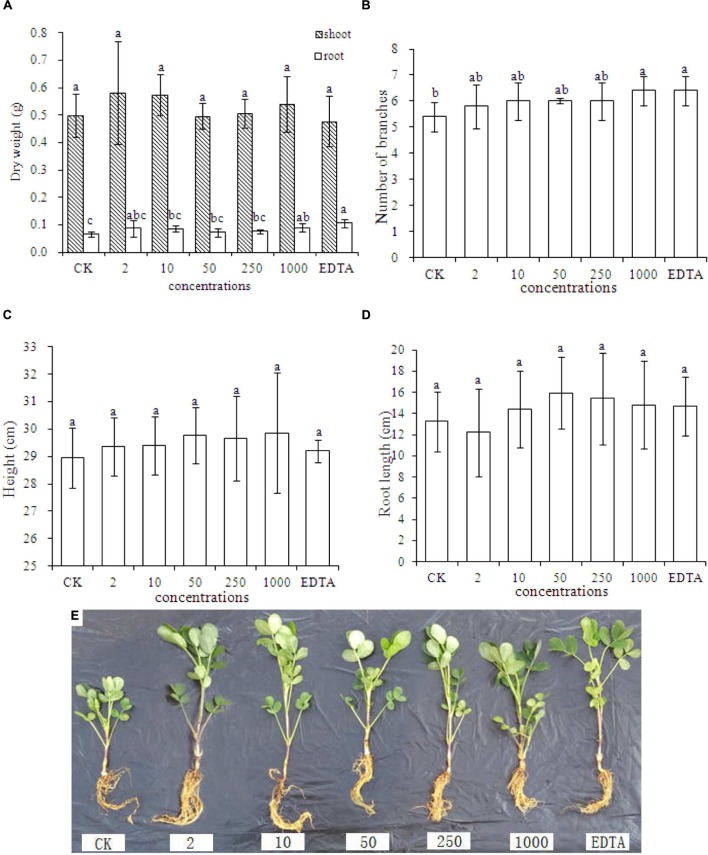
**Dry weight **(A)**, number of branches **(B)**, plant height **(C)**, root length **(D)**, and phenotypic image **(E)** of peanut seedlings grown in soil containing Fe_2_O_3_ NPs or EDTA-Fe fertilizer.** Error bars represent standard error (*n* = 3). Different letters represent significant differences among treatments (*p* < 0.05).

**Figures 2A–E** represent dry weight, number of branches, shoot height, root length, and phenotypic images of peanut treated with different concentrations of Fe_2_O_3_ NPs, respectively. As shown in **Figure 2A**, the significant increases of the root dry biomass were only evident in the treatment with 1000 mg⋅kg^-1^ Fe_2_O_3_ NPs and EDTA-Fe, whereas the root dry biomass did not display the statistical difference between these two treatments. In addition, no impact on biomass increases was found in the shoot dry biomass. This result is contrary as compared with the previous study by Liu et al. (2005), which demonstrated that Fe_2_O_3_ NPs could significantly elevate the dry biomass of peanut relative to the EDTA-Fe treatment. The plant height was higher in all Fe_2_O_3_ NPs treatments than EDTA-Fe and CK treatment, and the root length at high concentrations (50–1000 mg⋅kg^-1^) was longer than EDTA-Fe and CK treatment. Thus, Fe_2_O_3_ NPs could be an ideal substitution for EDTA-Fe in agriculture and can be utilized as a Fe source by peanut plants.

### SPAD Measurements of Peanut Plants

**FIGURE 3:**
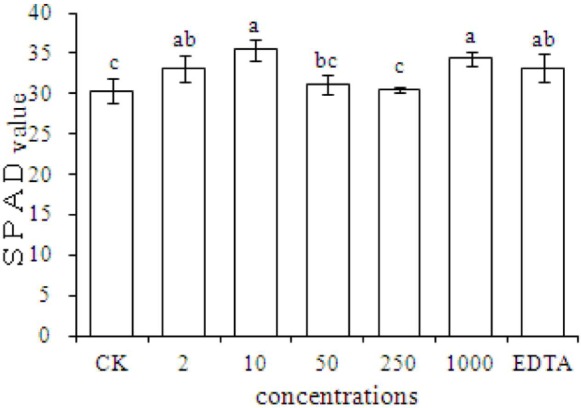
**SPAD measurements of peanut plants grown in soil without Fe (CK) or with EDTA-Fe and Fe_2_O_3_ NPs at various concentrations**.

Chlorophyll content is an important index of plant growth. The results showed that compared with the CK, the SPAD value significantly increased in plants grown with EDTA-Fe and with Fe_2_O_3_ NPs at dosages of 2, 10, and 1000 mg⋅kg^-1^ (**Figure 3**). The results also showed that peanut plant growth was promoted equally by Fe_2_O_3_ NPs and EDTA-Fe fertilizers.

Our results showed that Fe_2_O_3_ NPs increased the chlorophyll content in peanut plants. The chlorophyll contents in leaves of peanut plants were significantly higher in the 2, 10, 1000 mg⋅kg^-1^ Fe_2_O_3_ NPs treatments compared with the control group. Although the SPAD value in the EDTA-Fe treatment was notably increased, there was no difference between the Fe_2_O_3_ NPs and EDTA-Fe treatments. Therefore, Fe_2_O_3_ NPs treatments might be an ideal substitution for EDTA-Fe for peanut plant growth, as the evidence showed that Fe_2_O_3_ NPs could enhance the SPAD at the concentration as low as 2 mg⋅kg^-1^.

### Activities of Antioxidant Enzymes in Peanut Plants

**FIGURE 4:**
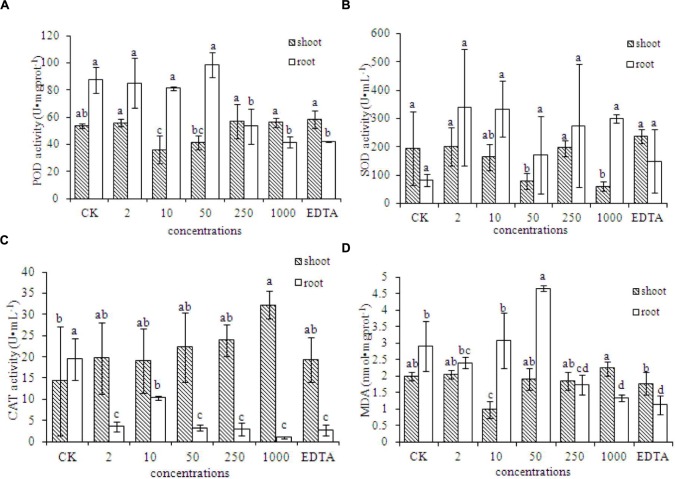
**Activities of antioxidant enzymes in the shoot and root of peanut plants grown without Fe (CK) and with EDTA-Fe fertilizers and Fe_2_O_3_ NPs.** **(A–C)** represent POD, SOD, CAT activities in shoot and root of peanut treated with different concentrations of Fe_2_O_3_ NPs. **(D)** is the MDA content in Fe_2_O_3_ NPs treated peanut.

The MDA content in the roots of peanut plants was significantly lower in the EDTA-Fe and two Fe_2_O_3_ NPs treatments (250 and 1000 mg⋅kg^-1^), compared with that in the control. However, the MDA contents in the shoots did not differ significantly among the treatments. Antioxidant enzymes showed different responses to Fe_2_O_3_ NPs and EDTA-Fe fertilizers (**Figures 4A–C**). In **Figure 4B**, superoxide dismutase (SOD) activities in both Fe_2_O_3_ NPs and EDTA-Fe treated peanut roots were higher than the control, however, due to the relative huge variance among the replicates, the statistical analysis showed otherwise. The presences of Fe_2_O_3_ NPs had no impact on the MDA content in peanut root at low exposure concentrations, except an exception at 50 mg⋅kg^-1^, which caused significantly high level of MDA as compared to the control (**Figure 4D**). Interestingly, as increasing the concentrations to 250 and 1000 mg⋅kg^-1^, the MDA content was notably decreased. The similar results were also evident in the EDTA-Fe treatment. The activities of peroxidase (POD) and catalase (CAT) were significantly lower in the EDTA-Fe and Fe_2_O_3_ NPs treatments than in the control (**Figures 4A,C**). In peanut shoots, CAT activity was higher in the EDTA-Fe treatment and all Fe_2_O_3_ NPs treatments than in the control, but the changes in POD and SOD activity did not show consistent trends in response to the EDTA-Fe fertilizers and Fe_2_O_3_ NPs.

### Phytohormone Contents in Peanut Plants

**FIGURE 5:**
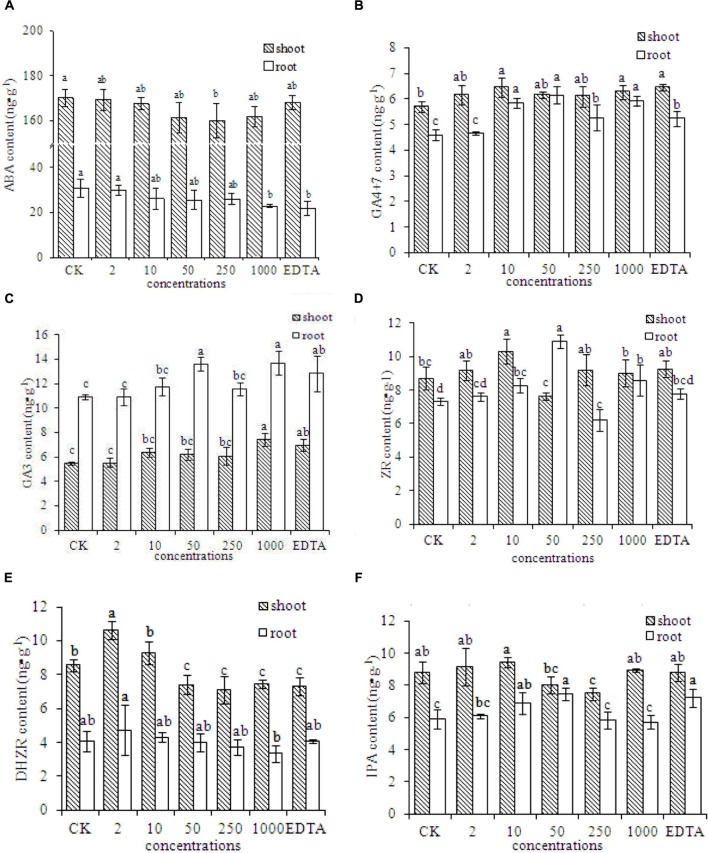
**Phytohormone contents in peanut plants grown without Fe (CK) or with Fe_2_O_3_ NPs or EDTA-Fe fertilizer.** The contents of phytohormone including ABA, GA4+7, GA3, ZR, DHZR, and IPA, are shown in **(A–F)**, respectively.

The contents of several plant hormones were evaluated in peanut plants grown without Fe (CK) or with EDTA-Fe fertilizers or Fe_2_O_3_ NPs. The ABA contents in peanut plants were lower in the EDTA-Fe and Fe_2_O_3_ NPs treatments than in the control (**Figure 5A**). The ABA concentrations were lower in roots than in shoots. The ABA content decreased as the Fe_2_O_3_ NPs concentration increased.

The concentrations of GA4+7 in roots and shoots of peanut were higher in the EDTA-Fe and Fe_2_O_3_ NPs treatments than in the control (**Figure 5B**). The GA3 contents were higher in the roots than in the shoots, and were generally higher in the EDTA-Fe and Fe_2_O_3_ NPs treatments than in the control (**Figure 5C**). The highest ZR contents in the shoots and roots were in the 10 and 50 mg⋅kg^-1^ Fe_2_O_3_ NPs treatments, respectively (**Figure 5D**). Compared with the control, the higher-concentration Fe_2_O_3_ NPs treatments (50–1000 mg⋅kg^-1^) showed lower DHZR contents in shoots, and the lower-concentration Fe_2_O_3_ NPs treatments (2–10 mg⋅kg^-1^) had higher DHZR contents in shoots. The DHZR concentrations in roots did not differ significantly among the treatments (**Figure 5E**). The IPA contents in peanut roots and shoots did not show consistent trends with respect to EDTA-Fe and Fe_2_O_3_ NPs treatments (**Figure 5F**).

### Total Fe Content in Peanut Plants

**FIGURE 6:**
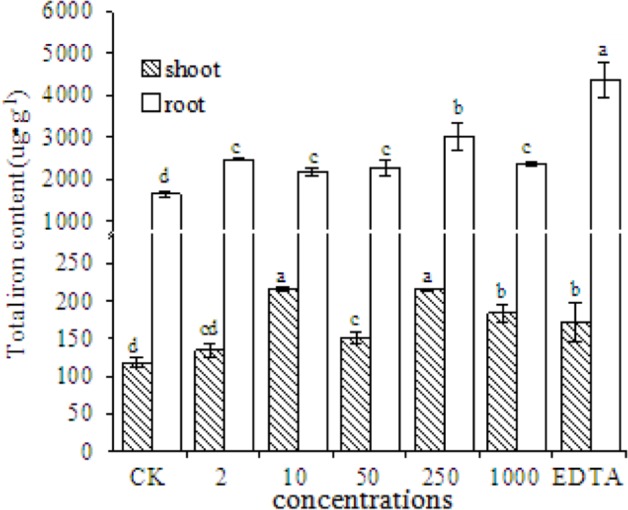
**Total iron content in shoots and roots of peanut plants grown without Fe (CK) or with EDTA-Fe fertilizers or Fe_2_O_3_ NPs**.

The total Fe content in the shoots and roots of peanut plants significantly increased in the EDTA-Fe and Fe_2_O_3_ NPs treatments (**Figure 6**). More Fe was taken up into roots than into shoots, probably because Fe is absorbed into plants via the roots. The Fe content in roots was higher in the EDTA-Fe treatment and Fe_2_O_3_ NPs treatments than in the control. The highest Fe content in shoots was in the 10 and 250 mg⋅kg^-1^ Fe_2_O_3_ NPs treatments (consistent with the SPAD results), followed by the 1000 mg⋅kg^-1^ Fe_2_O_3_ NPs and EDTA-Fe treatments.

### Scanning Electron Microscope Characterization of Fe_2_O_3_ NPs in Soil

**FIGURE 7:**
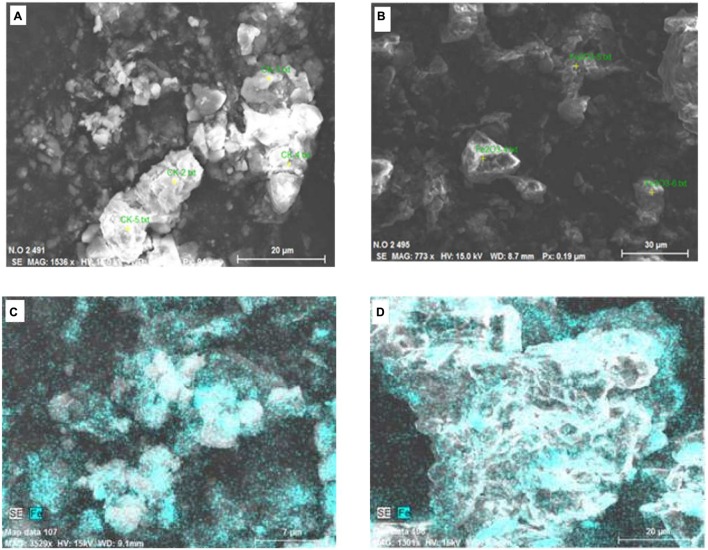
**Scanning electron microscope images of soil samples, **(A,C)** Control (no Fe); **(B,D)** Soil treated with Fe_2_O_3_ NPs at 1000 mg⋅kg^-1^**.

Soil samples from the control and the 1000 mg⋅kg^-1^ treatment were analyzed by SEM (**Figure 7**). Also, the element contents of particles in selected areas (yellow spots in images) were analyzed by EDS (**Figures 7A,B**). Soil is a complex system. The main components of the soil samples analyzed in this study were oxygen and silica (**Table 1**). In soil, Fe–Mn oxides are considered to be second most abundant mineral (Post, 1999), and Fe is present in small amounts. Iron was detected on soil particles in the control and Fe_2_O_3_ NPs treatments (**Figures 7A,B**). In **Figures 7C,D**, the green areas indicate Fe; the brighter the color, the higher the Fe content. The Fe contents in soil particles were markedly higher in the 1000 mg⋅kg^-1^ Fe_2_O_3_ NPs treatment than in the control (**Figures 7C,D**); 5.54% in control soil and 11.90% in 1000 mg⋅kg^-1^ Fe_2_O_3_ NPs treatment soil (**Table 1**). The Fe_2_O_3_ NPs in this study was charged. It is likely that Fe_2_O_3_ NPs in soil are adsorbed onto soil particles, and thereby retained in the soil matrix. Organic matter in the soil promotes the movement of Fe_2_O_3_ NPs, which can be absorbed and utilized by peanut plants.

**Table 1:** Mass percentage of detected elements in selected areas (yellow spots in **Figures 7A,B**).

Element	Mass % in CK	Mass % in nanoparticles1000 mg⋅kg^-1^
C	7.46	2.96
O	47.60	41.84
Na	0.39	0.41
Mg	1.70	2.70
Al	8.73	8.32
Si	19.53	20.10
K	6.46	2.96
Ca	2.51	6.49
Ti	0.09	2.31
Fe	5.54	11.90
**Total**	100	100


## Discussion

Physiological parameters including dry biomass, number of branches, height, root length, as well as phenotypic images, suggested that the additions of both Fe_2_O_3_ NPs and EDTA-Fe could elevate the peanut growth at the certain concentration (1000 mg⋅kg^-1^ for Fe_2_O_3_ NPs and 45.8 mg⋅kg^-1^ for EDTA-Fe in this study). Although the results of peanut height and root length also exhibit some increases at certain concentrations, the statistical analysis demonstrated otherwise because of huge variance among the replicates in each treatment. Taken together, Fe_2_O_3_ NPs were able to be used as a source of Fe by peanut plants and could be a promising substitution for EDTA-Fe.

The SPAD values are positively correlated with the chlorophyll content, and therefore, they are a reliable indicator of chlorophyll concentration (Vasconcelos and Grusak, 2014). Previous studies have shown that Fe deficiency leads to lower chlorophyll content, which is visible as chlorotic symptoms, and severely reduced plant yield (Mimmo et al., 2014; Sánchez-Alcalá et al., 2014; Vasconcelos and Grusak, 2014).

An excess or deficiency of Fe is harmful to peanut plants. Such nutrient stress conditions can lead to oxidative stress, which induces protection mechanisms to scavenge reactive oxygen species (Gui et al., 2015b). Based on the results of antioxidant enzyme activities and the MDA content, oxidative stress might not occur in the presence of either Fe_2_O_3_ NPs or EDTA-Fe with the indicated exposure doses. The additions of Fe_2_O_3_ NPs may stimulate reactive oxygen species (ROS) production in plants, which is also known as a signaling molecule and stimulate plant growth.

Plant hormones are organic substances synthesized in trace amounts by plants. They play important roles as signaling molecules to regulate many aspects of plant growth and development (Le et al., 2014; Davière and Achard, 2016). The phytohormone ABA inhibits growth, promotes senescence, and slows metabolism. The ABA contents in peanut plants were lower in the Fe_2_O_3_ NPs treatments than in the control. In previous studies, the ABA content of plants increased under nitrogen, phosphorous, potassium, zinc, and Fe deficiency (Radin, 1984; Lucini and Bernardo, 2015). Lower ABA contents can promote normal growth and delay senescence. Other plant hormones (e.g., IAA, IPA, GA, BR, and ZR) promote plant growth and delay senescence. Several previous studies have shown that the ABA content increased and the IAA and GA contents decreased under abiotic stress (Nadeem et al., 2010; Lucini and Bernardo, 2015; Davière and Achard, 2016). Our results were consistent with these findings; that is, the contents of GA4+7, GA3, and ZR were generally higher in Fe_2_O_3_ NPs treatments than in the control. Also, the GA4+7, GA3, and ZR contents were generally higher in the Fe_2_O_3_ NPs treatments than in the EDTA-Fe treatment.

The total Fe content observations revealed that the Fe contents in shoots corresponded to the SPAD results indicates that low concentrations of Fe_2_O_3_ NPs promoted the translocation of Fe from the roots to the shoots of peanut plants. Previous studies have demonstrated that metal-based NPs can accumulate and biotransform to other forms in plants. Due to their nano-effects, NPs is able to penetrate plant cell, which is different from the bulk NPs (in micrometer), and accumulate in plant tissues. Instead of analyzing the total Fe content, further study should focus on differentiation the Fe status, which can provide more information for Fe bio availability to plants.

Our results also demonstrated that a large amount of Fe_2_O_3_ NPs adhere to soil particles, thereby reducing nutrient loss. Previous studies have shown that electrons can accumulate at the edge of the soil particles (Bolland et al., 1976; Shuai and Yost, 2010). Peanut crops are mainly cultivated in northern China (Ding et al., 2011), in areas with sandy soils with low nutrient and organic matter contents (Kamolmanit et al., 2013; Jin et al., 2015). Such areas are susceptible to rapid changes in soil nutrient contents (Ulyett et al., 2014). Organic matter in soils can enhance the mobility of nanoparticles in porous media (Lin et al., 2010). It may be that the adsorption of Fe_2_O_3_ NPs to soil particles can improve their effectiveness as a Fe fertilizer.

In plants, Fe participates in photosynthesis, respiration, the biosynthesis of phytohormones and chlorophyll, and in electron transfer in redox reactions (Sánchez-Alcalá et al., 2014). Our results indicate that adding Fe_2_O_3_ NPs to the soil increased the biomass, chlorophyll content, and total Fe content of peanut plants. Overall, both Fe_2_O_3_ NPs and EDTA-Fe could notably increase peanut growth in terms of dry biomass and total chlorophyll content. The evidence for the reduction of antioxidant enzyme activities suggested that the additions of both types of Fe sources did not result in oxidative stress in plant, but stimulated the plant growth by producing the certain amounts of ROS, which is known as signaling molecules to trigger the root elongation and plant development.

## Conclusion

Fe_2_O_3_ NPs might be an ideal substitution for the traditional Fe fertilizer although the further studies are still needed to thoroughly assess its potential risk to the environmental and food security.

## Author Contributions

YR designed the experiment, MR carried the experiment and YH, JG, QZ, XF, ZZ, TH, and SZ helped MR do the experiment. YR, MR, XT, and CM wrote and revised manuscript.

## Conflict of Interest Statement

The authors declare that the research was conducted in the absence of any commercial or financial relationships that could be construed as a potential conflict of interest.
